# Supplementary material for: Contact tracing reveals community transmission of COVID-19 in New York City
Source: Nat Commun. 2022 Oct 23;13:6307. doi: 10.1038/s41467-022-34130-x (PMC9588776; doi:10.1038/s41467-022-34130-x)
Supplement: Supplementary file 1 — Supplementary Information [file 41467_2022_34130_MOESM1_ESM.pdf]

Supplementary Information

Contact tracing reveals community transmission of COVID-19 in New York City

Sen Pei[1]*, Sasikiran Kandula[1], Jaime Cascante Vega[1], Wan Yang[2], Steffen Foerster[3], Corinne Thompson[3], Jennifer Baumgartner[3], Shama Desai Ahuja[2,3], Kathleen Blaney[3], Jay K. Varma[4], Theodore Long[5], Jeffrey Shaman[1,6]

[1]Department of Environmental Health Sciences, Mailman School of Public Health, Columbia University, New York, NY, 10032, USA

[2]Department of Epidemiology, Mailman School of Public Health, Columbia University, New York, NY, 10032, USA

[3]New York City Department of Health and Mental Hygiene (DOHMH), Long Island City, NY, 11001, USA

[4]Department of Population Health Sciences, Weill Cornell Medical College, New York, NY, 10065, USA

[5]NYC Health + Hospitals, New York, NY

[6]Columbia Climate School, Columbia University, New York, NY, 10025, USA

[*]Correspondence to: S.P. (sp3449@cumc.columbia.edu)

Supplementary Methods

1. Data

The NYC Test & Trace Corps (T2) initiative attempts to interview all confirmed and probable COVID-19 patients about their location and activities before and during the infectious period. Information about close contacts during the infectious period is elicited during the interview. Close contacts are then interviewed and monitored for the duration of their quarantine. The program offers resources to support quarantine and isolation free of charge, including food delivery, medication delivery, and access to hotels. Close contacts are encouraged to get tested.

We used T2 data collected in NYC from October 1, 2020 to May 10, 2021. The study period spans the second pandemic wave of COVID-19 in NYC. The data contain 5,735,726 phone call records of interactions between contact tracers and confirmed/probable cases and their contacts, as well as information gathered during the phone calls. Age and zip code of home location are available for most cases and contacts. Index cases and their contacts were identified in the dataset using a matching algorithm based on personal identifying information (see below for details). We further cross-linked the contact tracing dataset with laboratory test results of COVID-19 in NYC during the same period to obtain the infection status of exposed persons. The daily numbers of contacted infected individuals in five age groups (0-9, 10-19, 20-49, 50-64, 65+) are shown in Supplementary Fig. 1a. During the study period, the age structure of confirmed cases remained stable; age group 20-49 accounted for most infections (Supplementary Fig. 1b). Percentage of circulating SARS-CoV-2 variants in NYC from January 2 2021 to May 8 2021 is shown in Supplementary Fig. 2. The numbers of tested exposures of different types and the secondary attack rate in each exposure type are reported in Supplementary Fig. 3. Use of this dataset in this study was approved by Columbia University Institutional Review Board (IRB) AAAT2182.

We used a two-step matching process to match individuals between the T2 contact tracing and DOHMH surveillance databases. In the first, heuristic step, pairwise matching was performed with blocking on any phone number and date of birth, requiring either of the two fields to be non-missing and both records to match exactly. Case-insensitive optimal string alignment distance was then computed between first names and last names of pairwise entries of blocked records to detect minor misspellings. Diminutive first names were flagged using text searching. String distance thresholds of two or less for the last name and one or zero or a diminutive flag for the first name were used to identify positive matches. If matched pairs from this heuristic matching process had non-matching person identifiers from the DOHMH and T2 databases or person identifiers were missing for at least one of the paired records, records entered a second matching step. For this step, an XGBoost model was trained using the following features: 1) Jaro-Winkler string distance score for first and last name; 2) optimal string alignment distance between phone numbers, allowing for transposition between home and mobile phones; when missing, 10 was imputed; 3) the count of month/date/year components of the dates of birth that matched exactly, accounting for the possibility of components being mis-entered into the database; when missing, 3 was used; 4) a flag to identify the transposition of month and date components of the dates of birth; 5) the difference in birth years as an absolute value; when missing, a difference of 90 was imputed; 6) the probability distribution of the first and last name;

7) optimal string alignment distance between normalized mailing addresses and zip codes; when missing, they were assumed to be different and a distance of 40 was imputed for mailing address and 5 for zip code. All predictors were standardized to z-scores. A gold-standard data set was created by subjectively hand-labeling a random sample of 2,886 step 2 input matches as good/bad matches based on the personally identifying information. The XGBoost model was trained and tuned using five-fold cross validation on an 80/20 training/test split, with stratification on the binary (good/bad match) target variable. The Area Under the Curve (AUC) was maximized at 0.937 with the following hyperparameters: nrounds = 10, max_depth = 6 and eta=0.3. We reviewed sensitivity, specificity and F1 scores on the test dataset at different predicted probability cut-off values to identify the threshold above which record pairs were automatically matched. We selected a predicted probability of a match of 0.5 as the final threshold; this choice was based on a near-maximum F1 score at this threshold, and because it is widely used in machine learning applications as the threshold of choice. The sensitivity at this cut-off was 0.957, specificity was 0.702 and precision was 0.922. Step 2 input matches that were classified as "good" matches moved on to the final matches and combined with step 1 matches. Final matches were joined to each other to turn a = b and b = c pairs into a = b = c relationships and assigned person IDs. Records that did not make it to the final matches list were treated as people represented by only one record and were each assigned a unique person ID.

Demographic and socioeconomic data for NYC zip code tabulation areas (ZCTA) were compiled from the 5-year American Community Survey (ACS) (https://www.census.gov/programs-surveys/acs/data.html). Variables include population size, population density (persons per square kilometer), percentage of Black residents, percentage of Hispanic residents, percentage of population over 65 years old, median household income, percentage of residents with bachelor's degree, and mean household size. We downloaded the 2019 estimates for these variables using the R package tidycensus[1].

COVID-19 surveillance data in NYC at the MOZCTA (modified ZIP code tabulation area) level are available at the GitHub repository maintained by the NYC Department of Health and Mental Hygiene (DOHMH) (https://github.com/nychealth/coronavirus-data). We used weekly cases per capita, weekly tests per capita, and percentage of tests positive. Vaccination data were obtained from the public repository of DOHMH (https://github.com/nychealth/covid-vaccine-data). Human mobility data recording the weekly number of visitors to points of interest (POIs) in NYC were provided by SafeGraph (https://safegraph.com/), which aggregates anonymized location data from numerous mobile phone applications to provide insights about physical places, via the SafeGraph Community. To enhance privacy, SafeGraph excludes census block group information if fewer than five devices visited an establishment in a month from a given census block group. We aggregated the mobility data to zip code level to estimate the weekly number of visitors (regardless of visitors' location of residence) to POIs in each zip code area. In the statistical analysis, we mapped the ACS data from the ZCTA level to the MOZCTA level to align the scale of the data. The mapping between ZCTA and MOZCTA is available at https://data.cityofnewyork.us/Health/Modified-Zip-Code-Tabulation-Areas-MODZCTA-/pri4-ifjk.

2. Reconstructing transmission networks

Due to asymptomatic and pre-symptomatic shedding, the reporting dates of index cases and contacts cannot be used to determine the direction of transmission. To address this issue, we developed a maximum-likelihood method to reconstruct transmission chains based on the risk of COVID-19 spread across different age groups. This approach includes three steps:

1) Estimate the infection time using symptom onset date or specimen collection date. Use the estimated infection time to determine the direction of exposure and transmission.

2) Estimate the probability of transmission for exposures across age groups using test and trace data.

3) Sample an ensemble of possible transmission networks and select the one that maximizes the transmission likelihood.

2.1 Estimation of infection time

For each pair of index case and contact, we inferred the direction of exposure or transmission using estimated infection time. All index cases were confirmed infections, but only a proportion of contacts were tested. We therefore used exposure pairs for which both the index case and contact had been tested and excluded exposure pairs for which contacts had not been tested, as these contacts did not affect the observed transmission network. If the contact tested negative, the direction of exposure is from the index case to the close contact (i.e., the index case is the infector); however, if the contact tested positive, the direction of exposure is uncertain and must be estimated.

For symptomatic cases who reported symptom onset dates, the infection time was estimated using the distribution of the incubation period reported from previous studies. Incubation period is the time between infection and symptom onset. Here we used a Weibull distribution estimated based on detailed contact tracing data from Hunan province, China[2]. Specifically, the probability density function (PDF) for the incubation distribution is

$$p(x) = \frac{k}{\lambda}\left(\frac{x}{\lambda}\right)^{k-1} e^{-\left(\frac{x}{\lambda}\right)^{k}}, \qquad [1]$$

where the shape parameter $k = 1.58$ and the scale parameter $\lambda = 7.11$. To estimate infection time, we randomly drew incubation periods (in days) for symptomatic cases from this PDF [1]. We also tested a log-normal incubation distribution estimated using contact tracing data from Shenzhen, China[3]. The transmission network remained similar.

Previous studies indicate that incubation period estimated using contact tracing data may be subject to bias depending on outbreak dynamics and the method used to calculate the delay distribution[4]. Typically, within-individual delay distributions such as the incubation period are not affected by epidemic dynamics if a forward delay distribution is used (i.e., a distribution of incubation period from a cohort of infected individuals that were infected at the same time).

However, the incubation period could be biased if a backward delay distribution is used (i.e., a distribution of incubation period from a cohort of infected individuals that developed symptoms at the same time). Specifically, the backward incubation period is biased low (high) when the epidemic is growing (declining). To reduce the effect of such potential bias on the network reconstruction, we used the incubation period distribution estimated in Hu et al[2]. In this study, the authors argued that the contact tracing data were collected from in-depth epidemiological investigations, allowing robust estimation of key time-to-event distributions. Moreover, the exponential growth phase of the outbreak lasted only about two weeks (thus the potential underestimation of incubation period is limited) and the effort heavily relied on forward contact tracing. The effect of the potential bias of incubation period on network reconstruction is therefore limited.

To verify the robustness of inference, we performed sensitivity analyses using two alternative incubation period distributions – one with a 10% underestimation of the mean ($k = 1.58$, $\lambda = 6.40$, mean 5.76 days) and another one with a 10% overestimation of the mean ($k = 1.58$, $\lambda = 7.82$, mean 7.04 days). We used the same shape parameter k and varied the scale parameter λ to adjust the mean incubation period. For each pair of index case and reported exposure with symptom onsets, we drew 1,000 samples of infection times using the incubation period distribution. Denote $p_{\rightarrow}$ as the fraction of samples that the inferred transmission direction is from the index case to exposure. We computed $\{p_{\rightarrow}\}$ for all pairs and calculated the change of $\{p_{\rightarrow}\}$ due to biased incubation period distributions. For the underestimated incubation period, the median change is -0.003 (95% CI: [-0.049, 0.046]); for the overestimated incubation period, the median change is 0.003 ([-0.044, 0.047]). This experiment indicates that potential bias in the incubation period distribution does not dramatically affect the inference of transmission direction.

For cases without symptoms, we used specimen collection date to estimate infection date. Denote $t_{inf\rightarrow test}$ as the interval from infection to specimen collection date. We aim to estimate $t_{inf\rightarrow test}$ given a person tested positive, i.e., $P\left(t_{inf\rightarrow test}\middle|positive\right)$. Using Bayes' rule, we have the following relation:

$$P\left(t_{inf\rightarrow test}\middle|positive\right) \propto P\left(t_{inf\rightarrow test}\right)P\left(positive\middle|t_{inf\rightarrow test}\right). \qquad [2]$$

Here $P\left(t_{inf\rightarrow test}\right)$ is the prior and $P\left(positive\middle|t_{inf\rightarrow test}\right)$ is the likelihood of testing positive given that specimens were collected $t_{inf\rightarrow test}$ days after infection.

The prior $P\left(t_{inf\rightarrow test}\right)$ can be approximated using the interval from infection to specimen collection date for symptomatic cases, which provides a roughly plausible range of $t_{inf\rightarrow test}$. For each tested symptomatic case, $t_{inf\rightarrow test}$ is the sum of the sampled incubation period (from infection to symptom onset) and the time from symptom onset to specimen collection date, available in the dataset. The distribution of the prior $P\left(t_{inf\rightarrow test}\right)$ is shown in Supplementary Fig. 4a.

The likelihood $P\left(positive\middle|t_{inf\rightarrow test}\right)$ was estimated using viral dynamics and limits of detection (LOD) for PCR tests. Following Larremore et al.[5], we generated synthetic viral dynamics in

infected persons. The log-transformed viral load (copies/mL), V, is a piece-wise linear function of the number of days after infection, t. Specifically, the viral dynamics is determined by three control points: $(t_0, 3)$, (t_{peak}, V_{peak}), and $(t_f, 6)$. Here t_0 is the time when log viral load reaches 3; t_{peak} is the peak timing of log viral load; V_{peak} is the peak magnitude of log viral load; and t_f is the time when log viral load falls to 6. The mean log viral load was computed using the following function:

$$\bar{V}(t) = \begin{cases} \frac{3t}{t_0}, t \le t_0 \\ 3 + \frac{(V_{peak} - 3)(t - t_0)}{t_{peak} - t_0}, t_0 < t \le t_{peak} \\ \max\left(V_{peak} - \frac{(V_{peak} - 6)(t - t_{peak})}{t_f - t_{peak}}, 0\right), t > t_{peak} \end{cases} \quad [3]$$

In simulations, the following parameter distributions were used: $t_0 \sim U(2.5, 3.5)$, $V_{peak} \sim U[7,11]$, $t_{peak} \sim \min(t_0 + 0.5 + \Gamma(1.5,1), 3)$, and $t_f \sim t_{peak} + U(2,6)$. Here $U(a, b)$ is a uniform distribution between a and b; and $\Gamma(a, b)$ is a Gamma distribution with a shape parameter a and a scale parameter b. The simulated log viral load on each day t was drawn from a Gaussian distribution:

$$V(t) \sim N(\mu = \bar{V}(t), \sigma^2 = 0.04\bar{V}(t)^2). \quad [4]$$

Sample trajectories of viral load are shown in Supplementary Fig. 4b.

We simulated 10^5 viral load trajectories. For each trajectory, we randomly drew a LOD from $U[2,3.5]$, defined as the threshold for positive results – the test result is positive if $V(t)$ is above the LOD and negative otherwise. Using simulated viral dynamics and LOD, we obtained the likelihood $P(positive|t_{inf\to test})$ as shown in Supplementary Fig. 4c.

We computed the posterior distribution $P(t_{inf\to test}|positive)$ using Eq. [2] (Supplementary Fig. 4d). The infection time for cases without symptoms was estimated using the distribution $P(t_{inf\to test}|positive)$. Once the infection time of index case - contact pairs had been sampled, using either symptom onset date or specimen collection date, the direction of exposure could be determined by the chronological order of infection.

2.2 Estimation of transmission probability across age groups

We further use the test and tracing date to estimate the transmission probability across age groups, which are used to reconstruct transmission chains. We classify the total population into four age groups: 0-9, 10-19, 20-64, and 65+. Denote $P_{a\to a'}(positive)$ as the probability of successful transmission for an exposure from age group a to a'. In actuality, we only observe $P_{a\to a'}(positive|test)$ among tested exposures. Bayes' rule gives

$$P_{a \to a'}(positive) = P_{a \to a'}(positive|test) \times \frac{P_{a \to a'}(test)}{P_{a \to a'}(test|positive)}, \quad [5]$$

where $P_{a \to a'}(test)$ is the probability that an exposure from age group a to a' is tested and $P_{a \to a'}(test|positive)$ is the probability of testing given a successful transmission from age group a to a', i.e. an infection. A diagram for Eq. [5] is provided in Supplementary Fig. 5.

If we assume the relative test-seeking probability between exposed and infected individuals is independent of age, then $P_{a \to a'}(test)/P_{a \to a'}(test|positive)$ is constant across age groups. We then can use the test positivity rate for exposure from age group a to a', $P_{a \to a'}(positive|test)$, to represent the relative transmission probability across age groups: $P_{a \to a'}(positive) = \gamma P_{a \to a'}(positive|test)$, where $\gamma = P_{a \to a'}(test)/P_{a \to a'}(test|positive)$. See Supplementary Fig. 5 for more details.

To compute $P_{a \to a'}(positive|test)$, we first used the method introduced in subsection 2.1 to determine the possible directions of exposure pairs for which both index case and contact were tested. Then we selected the pairs of exposures from age group a to a' and computed the probability of successful transmission for $a \to a'$ exposures. We repeated this analysis 1,000 times and took the average transmission probability $P_{a \to a'}(positive|test)$. Results are shown in Supplementary Table 1.

2.3 Reconstruction of the maximum likelihood transmission network

We combined the methods described in subsections 2.1 and 2.2 to reconstruct transmission networks. Using the method developed in subsection 2.1, we first estimated the possible directions of transmission events (in which both index case and contact tested positive) and used these directed transmission links to form a putative transmission network. For each transmission link ℓ, we identified the age groups for both patients (e.g., an exposure from age group a to a') and recorded the transmission probability across age groups for this link ℓ in Extended Data Table 1 (estimated in subsection 2. 2) as $P_\ell(positive|test)$. We computed the likelihood considering all transmission links: $L = \sqrt[n]{\prod_\ell P_\ell(positive|test)}$ where ℓ runs over all n transmission links in the network. We sampled 1,000 putative transmission networks and selected the network that maximizes the likelihood L among the ensemble of possible transmission networks.

3. Statistical analysis

We used conditional autoregressive (CAR) models to analyze non-household within- and cross-ZIP code transmission in two separate models. The CAR model was implemented in a Bayesian hierarchical framework. Specifically, we fitted a Poisson generalized linear mixed model (GLMM) where the random effect was modeled by CAR priors to account for the inherent spatial-temporal autocorrelation present in the disease transmission data.

3.1 Statistical model

We modeled the numbers of non-household within- and cross-ZIP code transmission events using a modified Poisson generalized linear mixed model. Denote $y_{within}(i,t)$ and $y_{cross}(i,t)$ as the weekly numbers of non-household within-ZIP code and cross-ZIP code transmission events in ZIP code i and week t. Here cross-ZIP code transmission events include both directions, i.e., transmission for which either infector or infectee lived in a certain ZIP code. The week for transmission is determined by the self-reported contact time between index cases and contacts. Fixed effects include log-transformed population density, log-transformed weekly cases per capita, log-transformed weekly tests per capita, cumulative cases per capita, percentage of Black residents, percentage of Hispanic residents, percentage of population over 65 years old, median household income, percentage of residents with a bachelor's degree, mean household size, percentage of fully vaccinated residents, and number of POI visitors per capita. All covariates were standardized to have mean zero and standard deviation one. We used log-transformed population as an offset, assuming the numbers of both within-ZIP code and cross-ZIP code transmission events are proportional to local population. In the regression model, we used the weekly case per capita to represent the local force of infection that impacts the number of observed within-ZIP code transmission events. While within-ZIP code transmission events are a subset of weekly cases, the observed transmission events are much fewer and may deviate from the pattern of weekly cases per capita due to reporting bias and other factors. For vaccination coverage, we observed a large spatial heterogeneity at the ZIP code level (Supplementary Fig. 6). The percentage of fully vaccinated population ranged from 22.7% to 82.8% in the week of May 22, 2021.

In the regression model, we used the raw number of POI visitors in each ZIP code area. The foot traffic data include information about the types of POIs, represented by the NAICS (North American Industry Classification System) code. However, there may be selection bias among POI categories. In addition, school-age children under 13 years old and other individuals without access to smart phones are not included in the data. Over the course of the pandemic, the change in POI visitation varied across different categories. For instance, the recovery for educational service was faster than for restaurant & bar and grocery & pharmacy (Supplementary Fig. 7, Restaurant & Bar (NAICS: 7224 (Drinking Places), 7225 (Restaurants and Other Eating Places)), Grocery & Pharmacy (NAICS: 445 (Food and Beverage Retailers), 456 (Health and Personal Care Retailers)), and Educational Service (NAICS: 61 (Educational Services))). Previous work has linked the differential change in POI visitation with socioeconomic status, finding that disadvantaged groups were not able to reduce their mobility as sharply, and that the POIs that they visit are more crowded and are therefore associated with higher risk[6].

Specifically, the model for non-household within-ZIP code transmission is described by the following equation:

$$
\begin{aligned}
log(y_{within}(i, t+d)) &= log(population(i)) + \beta_1 \times log(population\ density(i)) \\
&+ \beta_2 \times \log(weekly\ cases\ per\ capita(i,t)) \\
&+ \beta_3 \times \log(weekly\ tests\ per\ capita(i,t)) \\
&+ \beta_4 \times cumulative\ cases\ per\ capita(i,t) + \beta_5 \times\ \%\ Black\ resident(i) \\
&+ \beta_6 \times\ \%\ Hispanic\ resident(i) + \beta_7 \times\ \%\ resident\ over\ 65(i) \\
&+ \beta_8 \times\ median\ household\ income(i) + \beta_9 \times \%\ bachelor's\ degree(i) \\
&+ \beta_{10} \times mean\ household\ size(i) + \beta_{11} \times \%\ fully\ vaccinated\ resident(i,t) \\
&+ \beta_{12} \times weekly\ POI\ visitors\ per\ capita(i,t) + \psi_{it} + \varepsilon_{it}. \qquad [6]
\end{aligned}
$$

Here d is the lag (in weeks), $log(population(i))$ is the offset, ψ_{it} is the random effect for location i and week t, and ε_{it} is the error term. In the main model, we used $d = 0$ (no lag). We additionally tested $d = 1$ and $d = 2$ as a sensitivity analysis.

The model for cross-zip code transmission is defined similarly:

$$
\begin{aligned}
log(y_{cross}(i, t+d)) &= log(population(i)) + \beta_1 \times log(population\ density(i)) \\
&+ \beta_2 \times \log(weekly\ cases\ per\ capita(i,t)) \\
&+ \beta_3 \times \log(weekly\ tests\ per\ capita(i,t)) \\
&+ \beta_4 \times cumulative\ cases\ per\ capita(i,t) + \beta_5 \times\ \%\ Black\ resident(i) \\
&+ \beta_6 \times\ \%\ Hispanic\ resident(i) + \beta_7 \times\ \%\ resident\ over\ 65(i) \\
&+ \beta_8 \times\ median\ household\ income(i) + \beta_9 \times \%\ bachelor's\ degree(i) \\
&+ \beta_{10} \times mean\ household\ size(i) + \beta_{11} \times \%\ fully\ vaccinated\ resident(i,t) \\
&+ \beta_{12} \times weekly\ POI\ visitors\ per\ capita(i,t) + \psi_{it} + \varepsilon_{it}. \qquad [7]
\end{aligned}
$$

3.2 Controlling for spatial-temporal autocorrelation

We first fitted a model without considering spatial-temporal autocorrelation in the random effect ψ_{it}; however, we found signatures of spatial autocorrelation in the residuals using Moran's I test for each week. The dependent variables are also temporally autocorrelated. Neglecting such spatial-temporal autocorrelation will lead to overconfidence in the estimated effect size, i.e., the standard errors will be biased too small[7].

To account for this inherent spatial-temporal autocorrelation in the dependent variables $y_{within}(i,t)$ and $y_{cross}(i,t)$, we modeled the random effect ψ_{it} using conditional autoregressive (CAR) priors[8,9]. Specifically, we used the CAR model proposed by Rushworth et al.[10], which represents the spatial-temporal structure as a multivariate autoregressive process with a spatially autocorrelated precision matrix. The model is specified by[8]:

$$
\begin{gathered}
\psi_{it} = \phi_{it}, \qquad [8] \\
\boldsymbol{\phi}_t | \boldsymbol{\phi}_{t-1} \sim N(\rho_T \phi_{t-1}, \tau^2 \boldsymbol{Q}(\boldsymbol{W}, \rho_S)^{-1}), \quad t = 2, \dots, T \\
\boldsymbol{\phi}_1 \sim N(\boldsymbol{0}, \tau^2 \boldsymbol{Q}(\boldsymbol{W}, \rho_S)^{-1}), \\
\tau^2 \sim Inverse - Gamma(a, b), \\
\rho_S, \rho_T \sim Uniform(0,1).
\end{gathered}
$$

In this model $\boldsymbol{\phi}_t = (\phi_{1t}, \dots, \phi_{Kt})$ is the vector of random effects for time t, which evolve over time via a multivariate first order autoregressive process with temporal autoregressive parameter ρ_T. The spatial autocorrelation is induced by the variance $\tau^2 \boldsymbol{Q}(\boldsymbol{W}, \rho_S)^{-1}$. The precision matrix $\boldsymbol{Q}(\boldsymbol{W}, \rho_S)$ depends on the spatial adjacency matrix $\boldsymbol{W}$ and the spatial autoregressive parameter ρ_S. We used a binary spatial adjacency matrix $\boldsymbol{W} = (w_{ij})$, where the entry w_{ij} is one if location i and location j share a common border and is zero otherwise. Additionally, $w_{ii} = 0$. The functional form of $\boldsymbol{Q}(\boldsymbol{W}, \rho_S)$ is given by[11]

$$\boldsymbol{Q}(\boldsymbol{W}, \rho_S) = \rho_S[diag(\boldsymbol{W}\mathbf{1}) - \boldsymbol{W}] + (1 - \rho_S)\boldsymbol{I}, \qquad [9]$$

where $\mathbf{1}$ is the $K \times 1$ vector of ones and $\boldsymbol{I}$ is the $K \times K$ identity matrix.

We implemented the model using the function ST.CARar in the R package CARBayesST[12]. Using a Bayesian hierarchical framework, model coefficients in Eqs. [6-7] and parameters in Eq. [8] were estimated using a Markov chain Monte Carlo (MCMC) algorithm[13]. We fitted the model using data from 177 MOZCTAs and 31 weeks ($K = 177, T = 31$). In the main model, we used $d = 0$ (no lag). We generated 420,000 MCMC samples for each coefficient/parameter and discarded the first 20,000 samples as the burn-in period. The remaining samples were subsequently thinned by 20 to reduce the autocorrelation of the Markov chain. In total, 20,000 MCMC samples were generated for each coefficient and parameter. The convergence of Markov chains was diagnosed using the convergence diagnostic proposed by Geweke[14]. The diagnostic statistics for all coefficients and hyperparameters were within the range (-1.96, 1.96), suggesting convergence of Markov chains.

We further evaluated the spatial-temporal autocorrelation in the model residuals to confirm that spatial and temporal structures had been annihilated. Specifically, we examined the spatial autocorrelation in residuals from the 177 locations in each week using Moran's I. The spatial autocorrelation of the residuals was absent for most weeks ($p > 0.05$) (Supplementary Fig. 8). Moran's I is a measure of spatial autocorrelation that characterizes the correlation in a signal among nearby locations in space. Specifically, it is defined as

$$I = \frac{N}{W} \frac{\sum_{i=1}^{N} \sum_{j=1}^{N} w_{ij}(x_i - \bar{x})(x_j - \bar{x})}{\sum_{i=1}^{N} (x_i - \bar{x})^2}, \qquad [10]$$

where N is the number of spatial units, x is the variable of interest (here, the residual in each ZIP code area), $\bar{x}$ is the mean of x, w_{ij} is a matrix of spatial weights with zeros on the diagonal ($w_{ii} = 0$), and W is the sum of all w_{ij} ($W = \sum_{i=1}^{N} \sum_{j=1}^{N} w_{ij}$). Here, we define w_{ij} as the adjacency matrix for all ZIP code areas, i.e., $w_{ij} = 1$ if locations i and j are contiguous, $w_{ij} = 0$ otherwise. Under the null hypothesis of no autocorrelation, the expected value of I is $E(I) = -1/(N - 1)$, which approaches zero as N approaches infinity. The p-value of the Moran's I test is obtained by comparing the computed I value with the null distribution.

For each location, we evaluated the temporal autocorrelation in the residuals using the Durbin-Watson test. Results indicate that temporal autocorrelation was not significant for most locations

($p > 0.05$) (Supplementary Fig. 9). Durbin-Watson test is used to detect the presence of autocorrelation at lag 1 in the residuals from a regression model. Define e_t as the residual at time t. The Durbin-Watson test statistics is

$$d = \frac{\sum_{t=2}^{T}(e_t - e_{t-1})^2}{\sum_{t=1}^{T} e_t^2}. \qquad [11]$$

Here T is the number of observations. The value of d ranges between 0 and 4. For large T, d is approximately equal to $2(1-\rho)$, where ρ is the sample autocorrelation of the residuals. $d = 2$ indicates no autocorrelation; $d < 2$ indicates a positive serial correlation; $d > 2$ indicates a negative serial correlation.

The effects of covariates are represented by the exponentiated coefficient, or the incidence rate ratio. The incidence rate ratio quantifies the multiplicative change in the number of transmission events if each covariate increases by one standard deviation, adjusting for all other covariates in the model. The distributions of incidence rate ratios were obtained from the 20,000 MCMC samples. The median, 95% CI, and p-values were derived from these empirical distributions. The estimated coefficients for the main model are shown in Supplementary Table 2.

A few sensitivity analyses were performed to assess the robustness of the results. First, we additionally tested one-week and two-week lags. The qualitative results remained similar (Supplementary Figs. 10-11). Second, we used another form of the random effect model proposed by Knorr-Held et al.[15]. In this model, the spatial-temporal variation in the data is decomposed into three components: an overall spatial effect common to all time periods, an overall temporal trend common to all spatial units, and a set of independent space-time interactions. See more details in Ref.[8]. The model was fitted using the function ST.CARanova in the R package CARBayesST. A zero-week effect lag was used and the MCMC setting was the same. Results hold as in the main model (Supplementary Fig. 12). Third, we tested whether the findings are robust to potential different response rates in contact tracing among age groups. Specifically, we randomly removed 50% of the contact tracing records reported by individuals <18 years old or >65 years old, representing the scenario that children and elderly are less likely to report their close contacts. Results remain similar to the main model (Supplementary Fig. 13).

Supplementary References

1. Walker, K., Herman, M. & Eberwein, K. tidycensus: Load US Census Boundary and Attribute Data as ‘tidyverse’ and ’sf’-Ready Data Frames. (2021).
2. Hu, S. *et al.* Infectivity, susceptibility, and risk factors associated with SARS-CoV-2 transmission under intensive contact tracing in Hunan, China. *Nat. Commun.* **12**, 1533 (2021).
3. Bi, Q. *et al.* Epidemiology and transmission of COVID-19 in 391 cases and 1286 of their close contacts in Shenzhen, China: a retrospective cohort study. *Lancet Infect. Dis.* **20**, 911–919 (2020).
4. Park, S. W. *et al.* Forward-looking serial intervals correctly link epidemic growth to reproduction numbers. *Proc. Natl. Acad. Sci.* **118**, (2021).
5. Larremore, D. B. *et al.* Test sensitivity is secondary to frequency and turnaround time for COVID-19 screening. *Sci. Adv.* eabd5393 (2020) doi:10.1126/sciadv.abd5393.
6. Chang, S. *et al.* Mobility network models of COVID-19 explain inequities and inform reopening. *Nature* **589**, 82–87 (2021).
7. F. Dormann, C. *et al.* Methods to account for spatial autocorrelation in the analysis of species distributional data: a review. *Ecography* **30**, 609–628 (2007).
8. Lee, D., Rushworth, A. & Napier, G. Spatio-Temporal Areal Unit Modeling in R with Conditional Autoregressive Priors Using the CARBayesST Package. *J. Stat. Softw.* **84**, 1–39 (2018).
9. Lee, D. CARBayes: an R package for Bayesian spatial modeling with conditional autoregressive priors. *J. Stat. Softw.* **55**, 1–24 (2013).

10. Rushworth, A., Lee, D. & Mitchell, R. A spatio-temporal model for estimating the long-term effects of air pollution on respiratory hospital admissions in Greater London. *Spat. Spatio-Temporal Epidemiol.* **10**, 29–38 (2014).
11. Leroux, B. G., Lei, X. & Breslow, N. Estimation of Disease Rates in Small Areas: A new Mixed Model for Spatial Dependence. in *Statistical Models in Epidemiology, the Environment, and Clinical Trials* (eds. Halloran, M. E. & Berry, D.) 179–191 (Springer, 2000). doi:10.1007/978-1-4612-1284-3_4.
12. Lee, D., Rushworth, A. & Pettersson, G. N. and W. CARBayesST: Spatio-Temporal Generalised Linear Mixed Models for Areal Unit Data. (2021).
13. Gelman, A., Carlin, J. B., Stern, H. S. & Rubin, D. B. *Bayesian Data Analysis*. (Chapman and Hall/CRC, 1995). doi:10.1201/9780429258411.
14. Geweke, J. Evaluating the Accuracy of Sampling-Based Approaches to the Calculation of Posterior Moments. in *In Bayesian Statistics* 169–193 (University Press, 1992).
15. Knorr-Held, L. Bayesian modelling of inseparable space-time variation in disease risk. *Stat. Med.* **19**, 2555–2567 (2000).

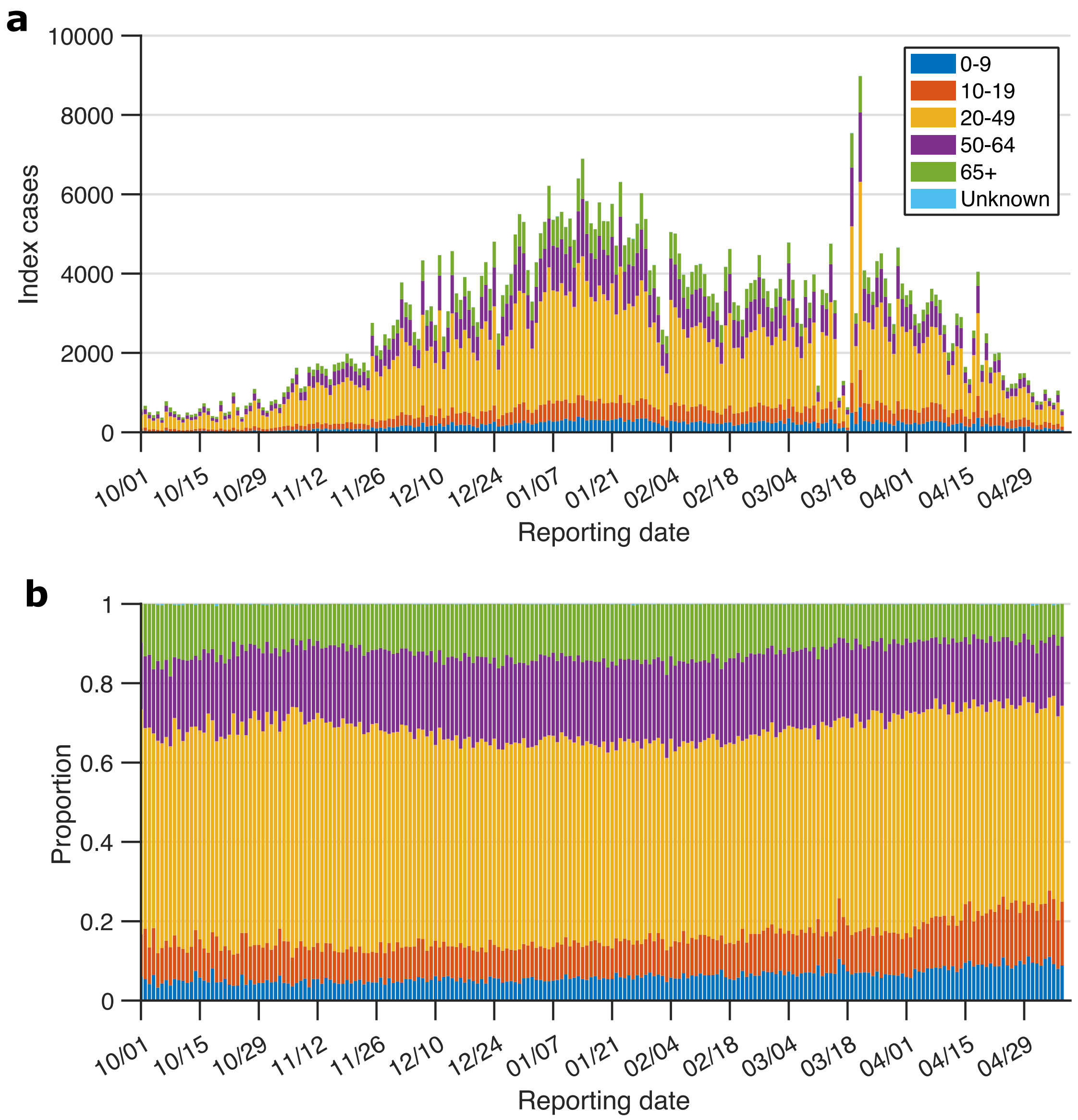

Supplementary Figure 1. Age structure of index cases called by contact tracers. (a) The daily number of index cases in each age group from October 1 2020 to May 10 2021. There was a data reporting issue during March 2021 so the spike in March does not reflect the actual COVID-19 situation. (b) The proportion of index cases in each age group during the study period.

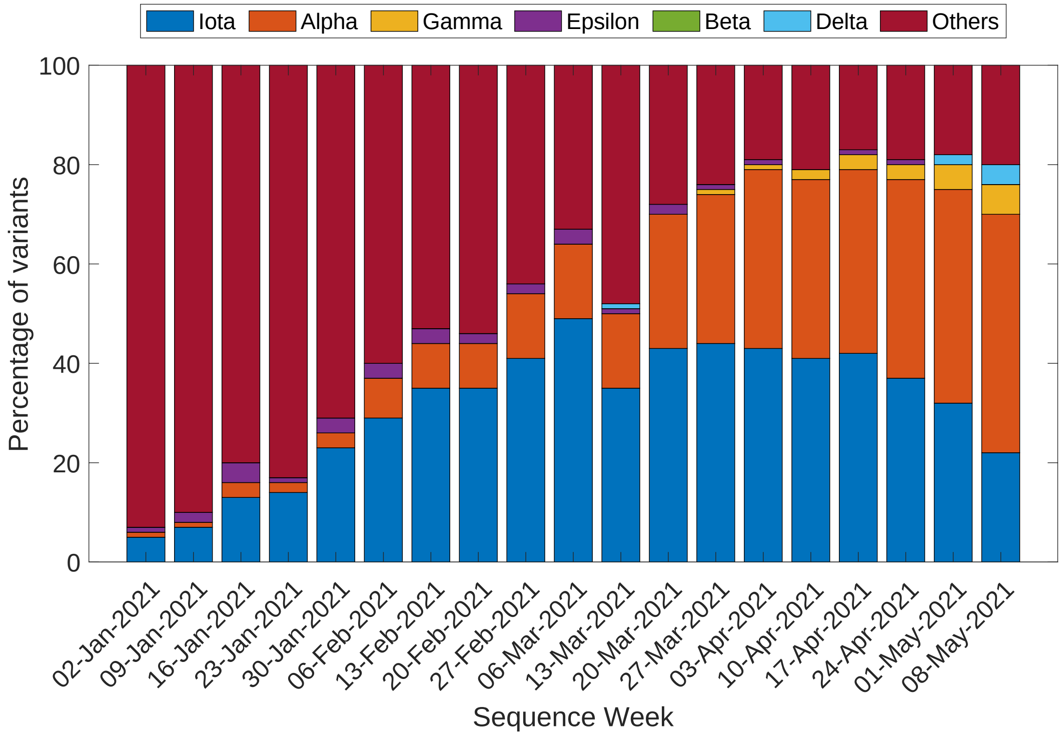

Supplementary Figure 2. Percentage of SARS-CoV-2 variants in NYC from January 2 2021 to May 8 2021. Prior to January 2 2021, the circulating strains in NYC were dominated by the index virus strain (categorized into "Others" in this figure). Data were obtained from the NYC DOHMH public repository (https://github.com/nychealth/coronavirus-data/tree/master/variants).

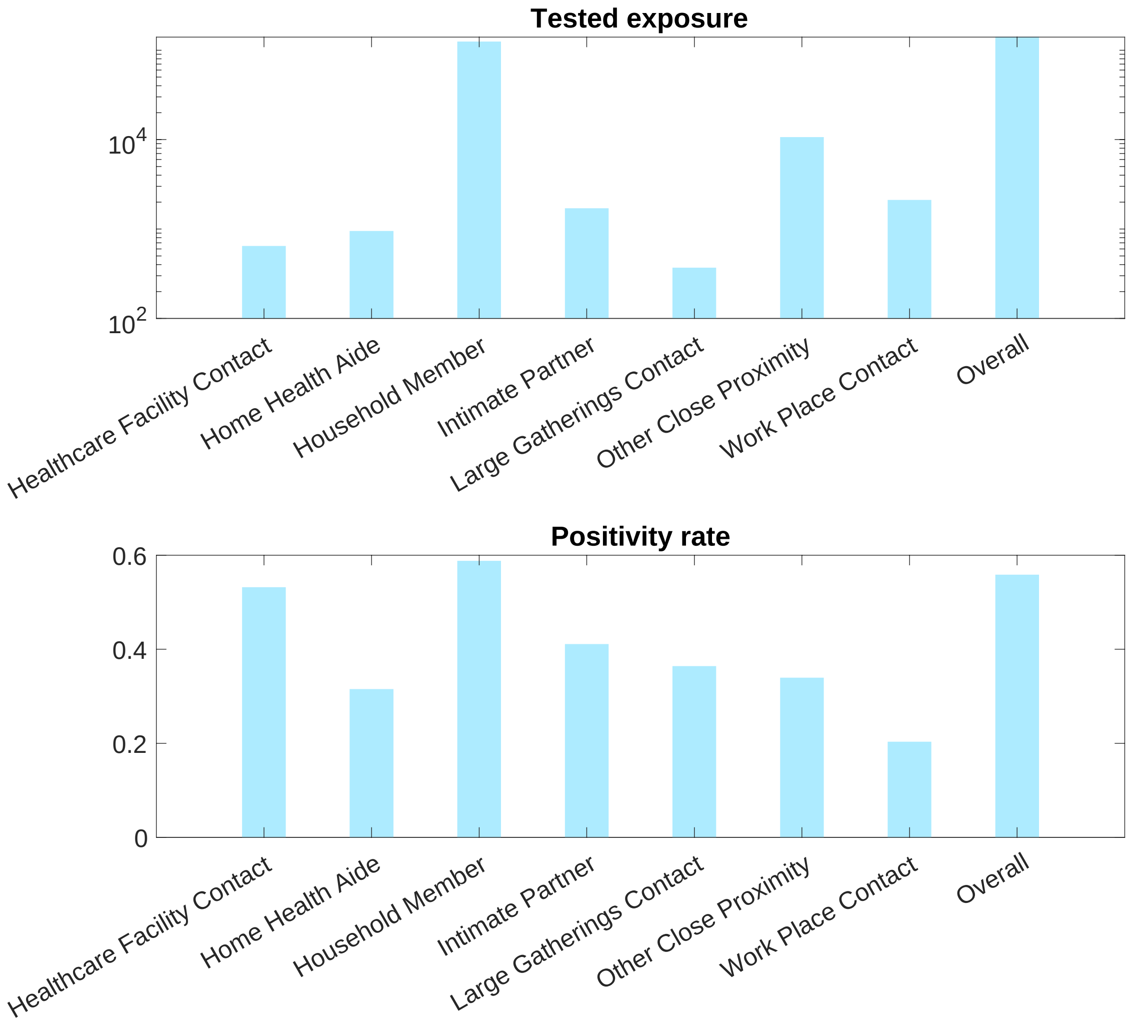

Supplementary Figure 3. Positivity rate among tested exposures. The numbers of tested exposures of different types are shown in the upper panel (log scale). Lower panel shows the positivity rate for each exposure type.

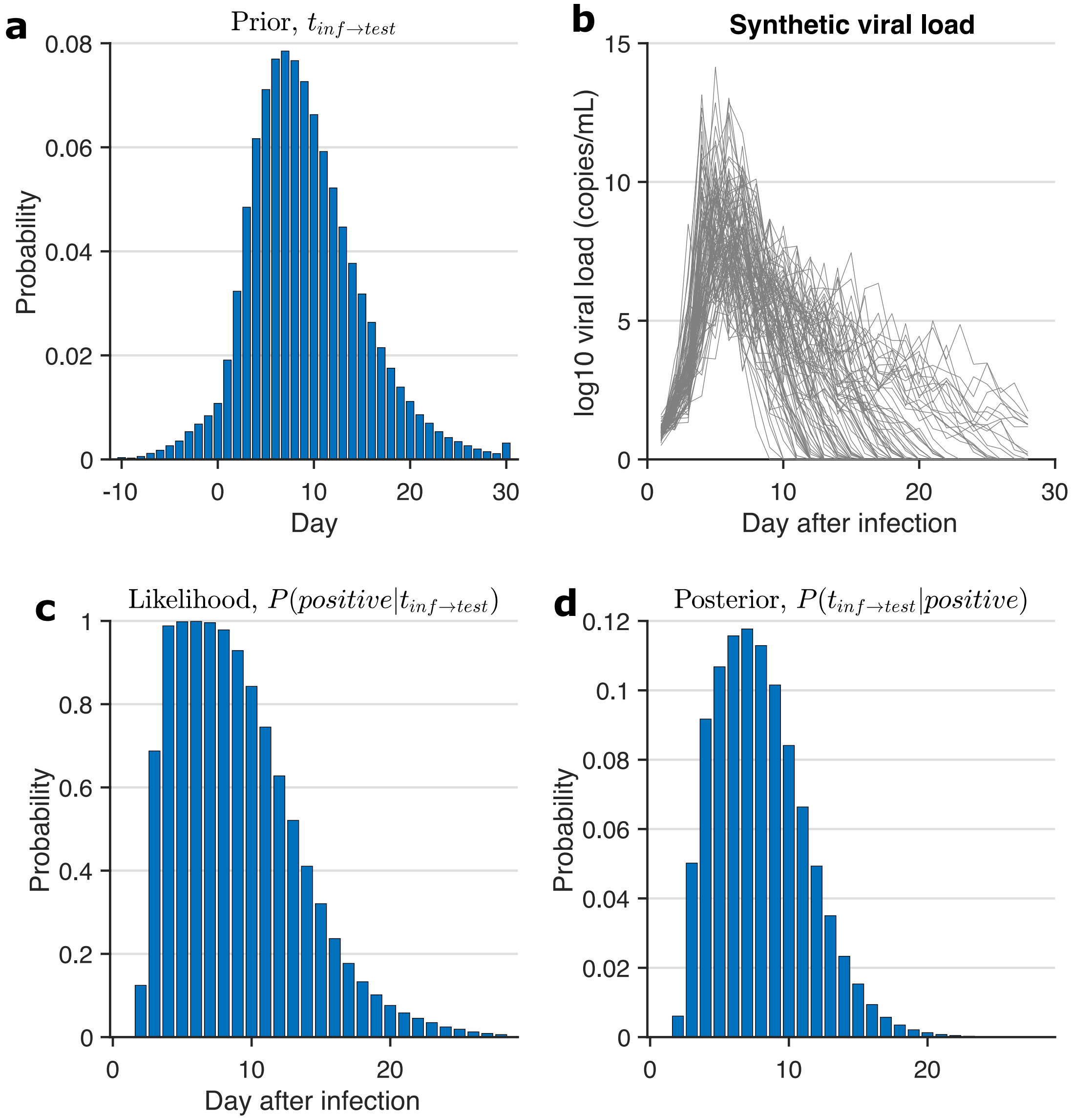

Supplementary Figure 4. Estimating the distribution of the interval from infection to testing for positive cases. (a) The prior distribution of $t_{inf \to test}$ obtained using data from symptomatic infections. (b) Samples of synthetic viral load trajectories. (c). The likelihood $P(positive|t_{inf \to test})$ obtained using synthetic viral dynamics and LOD. (d). The posterior $P(t_{inf \to test}|positive)$.

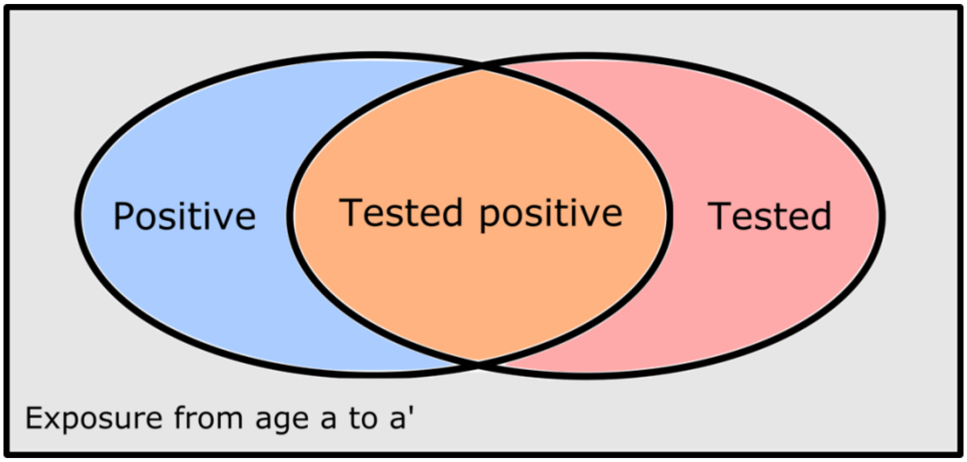

Supplementary Figure 5. A diagram for estimating the transmission probability for an exposure from age group a to a'. Assume the grey rectangular has an area of 1, representing all exposures from age group a to a'. The combined orange and red areas represent the probability of testing, $P_{a\to a'}(test)$; the combined orange and blue areas represent the probability of infection, $P_{a\to a'}(positive)$; the ratio of the orange area to the combined orange and red areas is the positivity rate, $P_{a\to a'}(positive|test) = orange/(orange + red)$; the ratio of the orange area to the combined orange and blue areas is the probability of testing among infected exposures, $P_{a\to a'}(test|positive) = orange/(orange + blue)$. Since the orange area can be computed by $P_{a\to a'}(test) \times P_{a\to a'}(positive|test)$, or $P_{a\to a'}(positive) \times P_{a\to a'}(test|positive)$, we have $P_{a\to a'}(test) \times P_{a\to a'}(positive|test) = P_{a\to a'}(positive) \times P_{a\to a'}(test|positive)$. Then we can estimate the transmission probability for an exposure from age group a to a' through $P_{a\to a'}(positive) = P_{a\to a'}(test) \times P_{a\to a'}(positive|test)/P_{a\to a'}(test|positive)$.

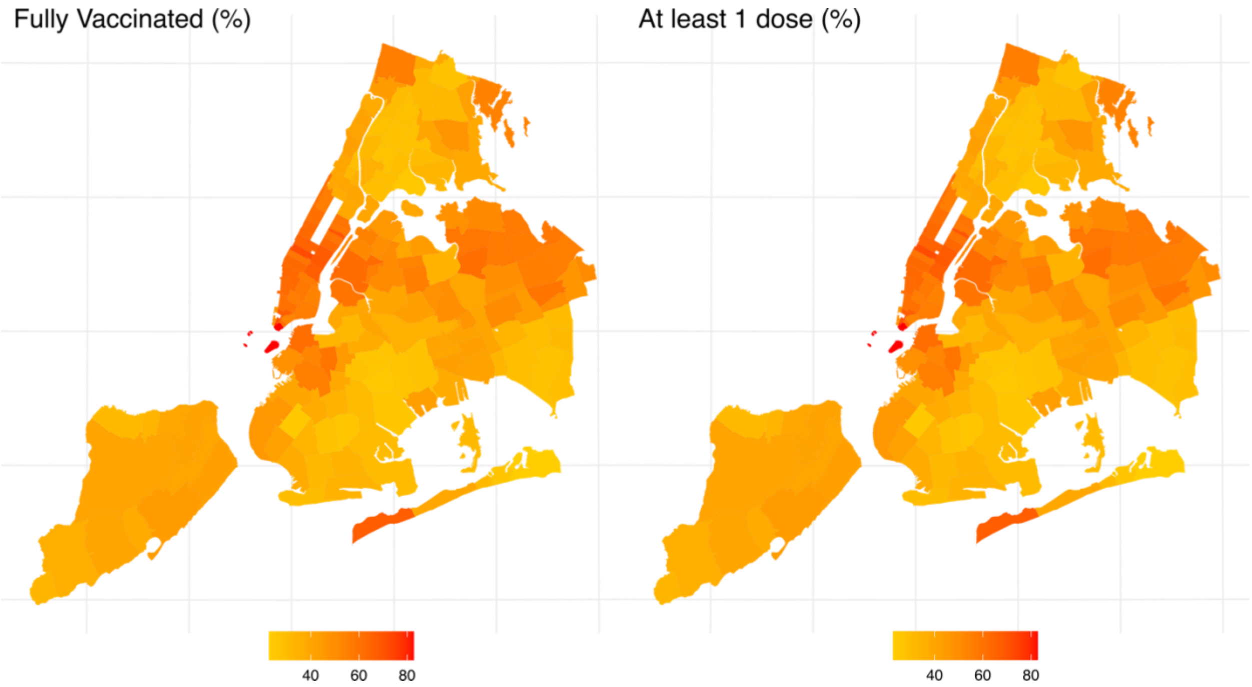

Supplementary Figure 6. Vaccination coverage at the ZIP code scale in NYC on May 22 2021. Percentages of fully vaccinated population (two doses) and those with at least one dose are shown in the left and right panel, respectively.

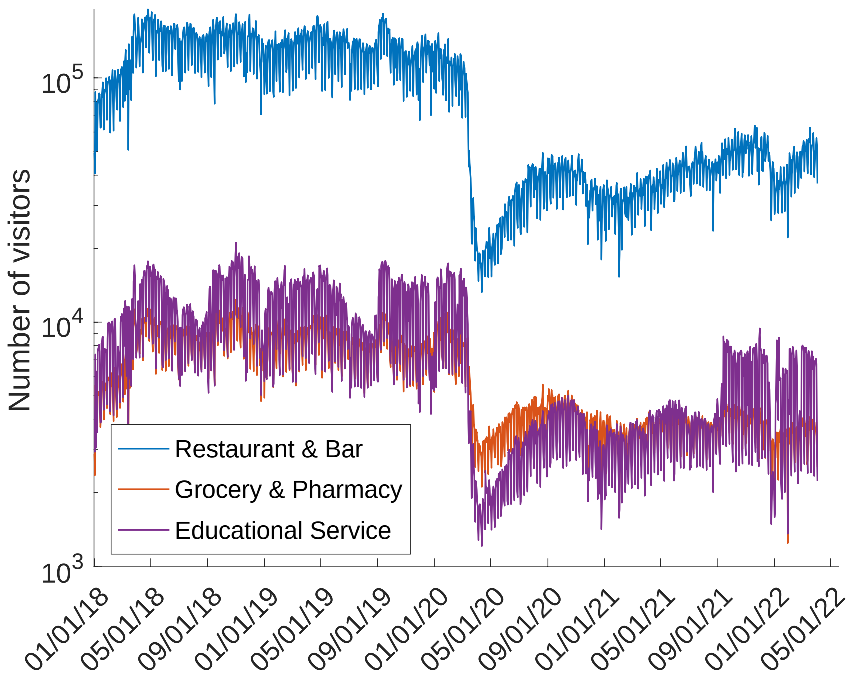

Supplementary Figure 7. The daily number of visitors for three categories of POIs: Restaurant & Bar (NAICS: 7224 (Drinking Places), 7225 (Restaurants and Other Eating Places)), Grocery & Pharmacy (NAICS: 445 (Food and Beverage Retailers), 456 (Health and Personal Care Retailers)), and Educational Service (NAICS: 61 (Educational Services)).

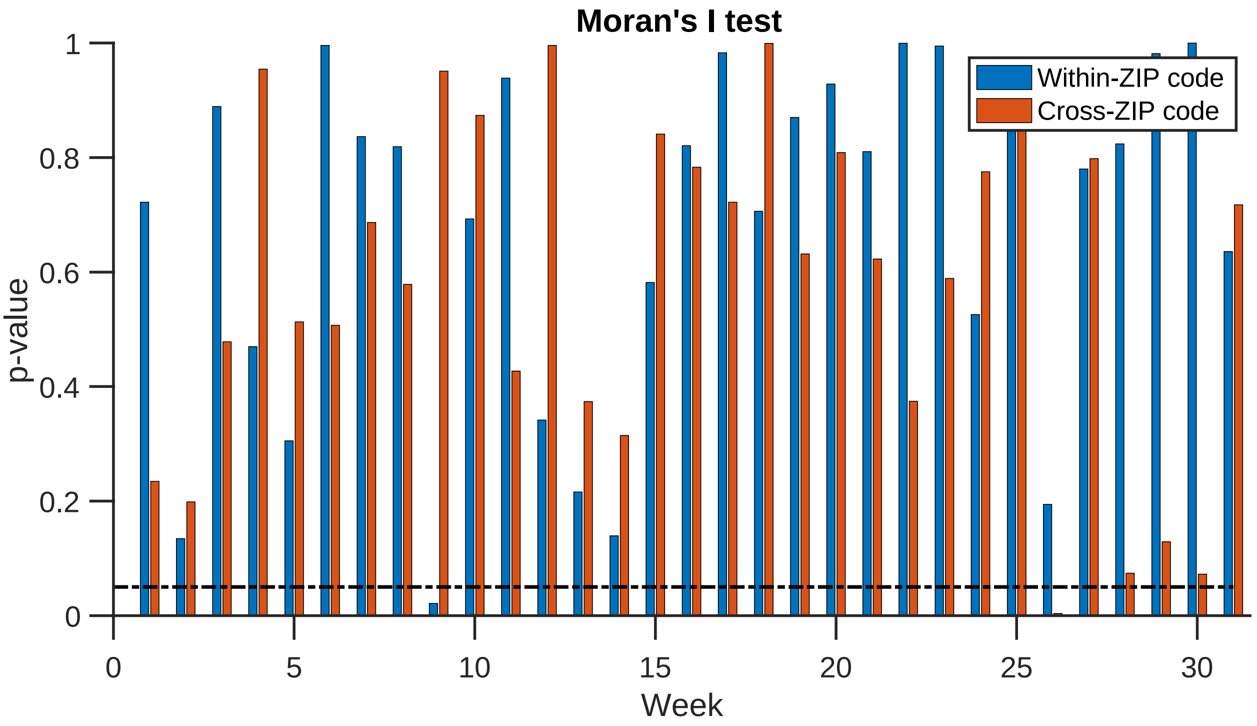

Supplementary Figure 8. Moran’s I test for residual spatial autocorrelation. Moran’s I test was performed for each week. P-values for within- and cross-zip code transmission are shown. The horizontal dash line marks the p=0.05 threshold. The Moran’s I test was two-sided and no adjustment was made for multiple comparisons.

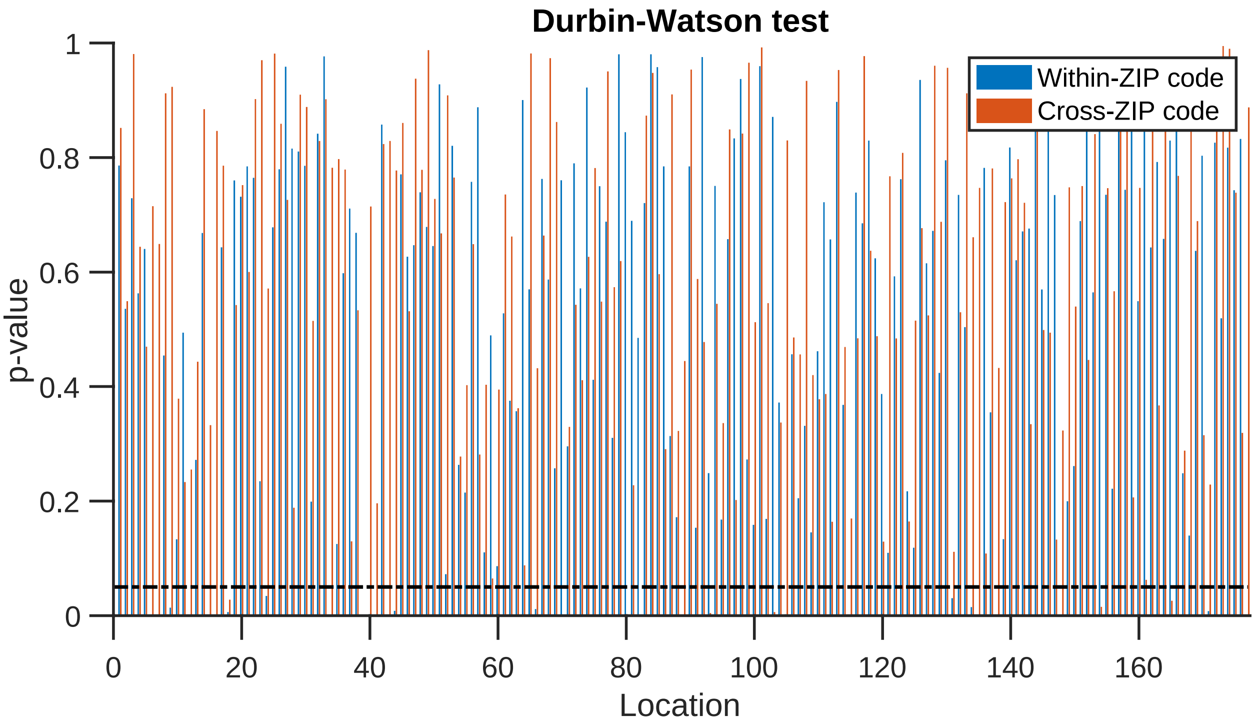

Supplementary Figure 9. Durbin-Watson test for residual temporal autocorrelation performed for each location. P-values for within- and cross-zip code transmission are shown. The horizontal dash line marks the p=0.05 threshold. The Durbin-Watson test was two-sided and no adjustment was made for multiple comparisons.

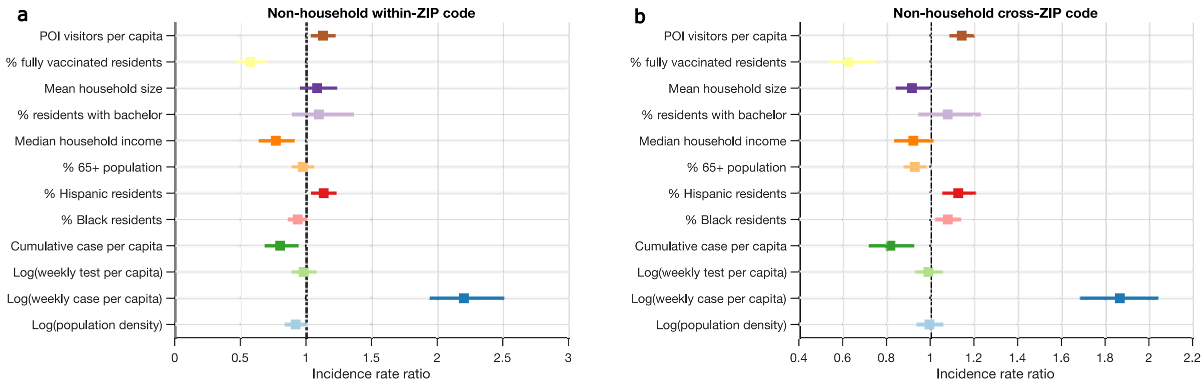

Supplementary Figure 10. Results of sensitivity analysis using a one-week lag. Dots and horizontal lines show median values and 95% CIs. DIC=6,301 for a and DIC=12,555 for b. Distributions in (a) and (b) were obtained using $n = 20{,}000$ MCMC samples of the posterior estimates.

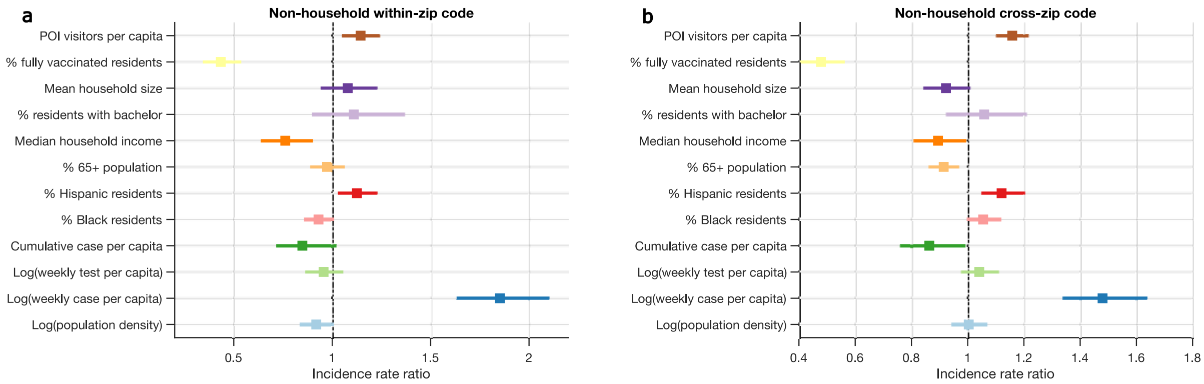

Supplementary Figure 11. Results of sensitivity analysis using a two-week lag. Dots and horizontal lines show median values and 95% CIs. DIC=6,257 for a and DIC=12,401 for b. Distributions in (a) and (b) were obtained using $n = 20{,}000$ MCMC samples of the posterior estimates.

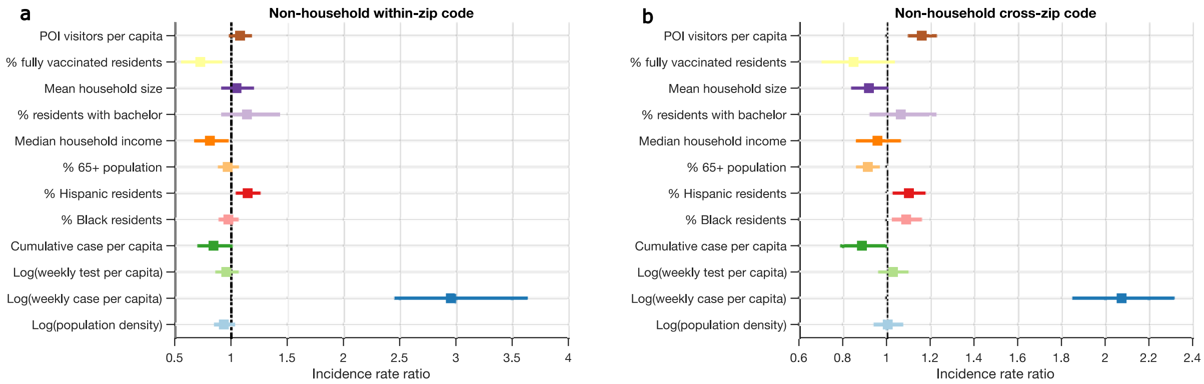

Supplementary Figure 12. Results of sensitivity analysis using an alternate random effects model form. No lag effect was used. Dots and horizontal lines show median values and 95% CIs. DIC=6,349 for a and DIC=12,641 for b. Distributions in (a) and (b) were obtained using $n = 20{,}000$ MCMC samples of the posterior estimates.

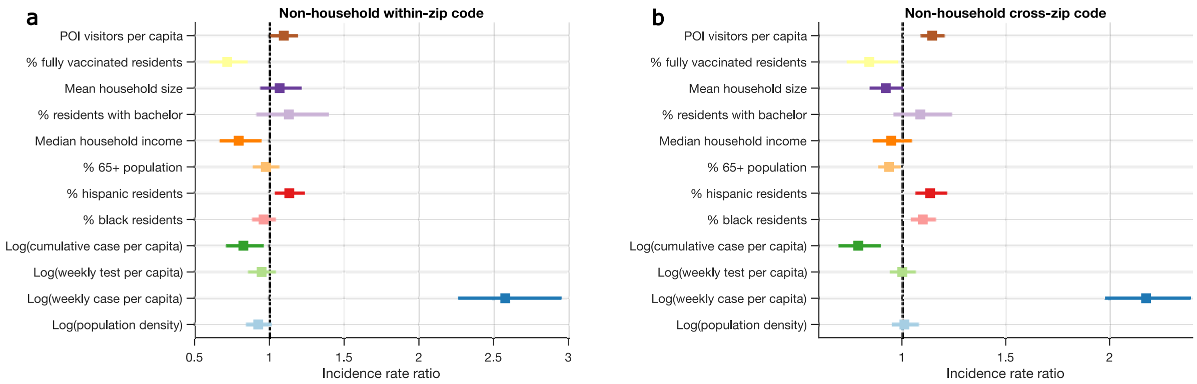

Supplementary Figure 13. Results of sensitivity analysis assuming 50% of index cases <18 years old and >65 years old do not report their close contacts. No lag effect was used. Dots and horizontal lines show median values and 95% CIs. DIC=6,340 for a and DIC=12,644 for b. Distributions in (a) and (b) were obtained using $n = 20{,}000$ MCMC samples of the posterior estimates.

	0-9	10-19	20-64	65+
**0-9**	0.507	0.499	0.457	0.516
**10-19**	0.469	0.472	0.436	0.530
**20-64**	0.470	0.440	0.346	0.482
**65+**	0.457	0.449	0.388	0.604

Supplementary Table 1. Transmission probability $P_{a \to a'}(positive|test)$ across age groups. Row indicates the age group of index cases, and column indicates the age group of contacts.

Variables	Non-household within-ZIP code	Non-household cross-ZIP code
POI visitors per capita	0.0914 (0.0031, 0.1765)	0.1346 (0.0839, 0.1842)
% fully vaccinated residents	-0.3281 (-0.5100, -0.1503)	-0.1600 (-0.3066, -0.0173)
Mean household size	0.0700 (-0.0573, 0.1981)	-0.0798 (-0.1684, 0.0077)
% residents with bachelor	0.1298 (-0.0660, 0.3394)	0.0852 (-0.0411, 0.2138)
Median household income	-0.2342 (-0.4034, -0.0714)	-0.0564 (-0.1564, 0.0432)
% 65+ population	-0.0218 (-0.1132, 0.0703)	-0.0640 (-0.1227, -0.0066)
% Hispanic residents	0.1286 (0.0405, 0.2149)	0.1244 (0.0562, 0.1946)
% Black residents	-0.0375 (-0.1207, 0.0438)	0.0947 (0.0398, 0.1527)
Cumulative case per capita	-0.1902 (-0.3448, -0.0364)	-0.2349 (-0.3671, -0.1116)
Log(weekly test per capita)	-0.0537 (-0.1506, 0.0426)	0.0022 (-0.0609, 0.0639)
Log(weekly case per capita)	0.9508 (0.8176, 1.0864)	0.7761 (0.6817, 0.8666)
Log(population density)	-0.0782 (-0.1695, 0.0165)	0.0107 (-0.0528, 0.0770)

Supplementary Table 2. Estimated coefficients for the main model. Results show the median estimates and 95% CIs. Significant covariates ($p<0.05$) are highlighted in blue. Two-sided Student’s t-test was used to determine the significance of coefficients.
