# Supplementary material for: Contact tracing reveals community transmission of COVID-19 in New York City
Source: Nat Commun. 2022 Oct 23;13:6307. doi: 10.1038/s41467-022-34130-x (PMC9588776; doi:10.1038/s41467-022-34130-x)
Supplement: Supplementary file 3 — Reporting Summary [file 41467_2022_34130_MOESM3_ESM.pdf]

nature research

Corresponding author(s): Sen Pei

Last updated by author(s): Oct 12, 2022

Reporting Summary

Nature Research wishes to improve the reproducibility of the work that we publish. This form provides structure for consistency and transparency in reporting. For further information on Nature Research policies, see our Editorial Policies and the Editorial Policy Checklist.

Statistics

For all statistical analyses, confirm that the following items are present in the figure legend, table legend, main text, or Methods section.

n/a	Confirmed	
☐	☒	The exact sample size (*n*) for each experimental group/condition, given as a discrete number and unit of measurement
☒	☐	A statement on whether measurements were taken from distinct samples or whether the same sample was measured repeatedly
☐	☒	The statistical test(s) used AND whether they are one- or two-sided<br>*Only common tests should be described solely by name; describe more complex techniques in the Methods section.*
☐	☒	A description of all covariates tested
☒	☐	A description of any assumptions or corrections, such as tests of normality and adjustment for multiple comparisons
☐	☒	A full description of the statistical parameters including central tendency (e.g. means) or other basic estimates (e.g. regression coefficient) AND variation (e.g. standard deviation) or associated estimates of uncertainty (e.g. confidence intervals)
☐	☒	For null hypothesis testing, the test statistic (e.g. *F*, *t*, *r*) with confidence intervals, effect sizes, degrees of freedom and *P* value noted<br>*Give P values as exact values whenever suitable.*
☒	☐	For Bayesian analysis, information on the choice of priors and Markov chain Monte Carlo settings
☒	☐	For hierarchical and complex designs, identification of the appropriate level for tests and full reporting of outcomes
☒	☐	Estimates of effect sizes (e.g. Cohen's *d*, Pearson's *r*), indicating how they were calculated

Our web collection on statistics for biologists contains articles on many of the points above.

Software and code

Policy information about availability of computer code

Data collection	No commercial software was used to collect data.
Data analysis	Data were analyzed using MATLAB R2021a, R statistical software version 4.1.0., R packages tidycensus (version 1.2.3) and CARBayesST (version 3.2.3). Custom code is available at https://github.com/SenPei-CU/NYC_contacttracing.

For manuscripts utilizing custom algorithms or software that are central to the research but not yet described in published literature, software must be made available to editors and reviewers. We strongly encourage code deposition in a community repository (e.g. GitHub). See the Nature Research guidelines for submitting code & software for further information.

Data

Policy information about availability of data

All manuscripts must include a data availability statement. This statement should provide the following information, where applicable:

- Accession codes, unique identifiers, or web links for publicly available datasets
- A list of figures that have associated raw data
- A description of any restrictions on data availability

COVID-19 surveillance data in NYC at the MOZCTA (modified ZIP code tabulation area) level are publicly available at the GitHub repository maintained by the NYC Department of Health and Mental Hygiene (DOHMH) (https://github.com/nychealth/coronavirus-data). Demographic and socioeconomic data for NYC zip code tabulation areas (ZCTA) are available from the 5-year American Community Survey (ACS) (https://www.census.gov/programs-surveys/acs/data.html). Contact tracing records and individual testing results are subject to restrictions for the protection of patient privacy. Requests for data access should be addressed to NYC DOHMH and NYC Health + Hospitals or the corresponding author. The corresponding author will respond to requests within two weeks and facilitate communications with NYC DOHMH and NYC Health + Hospitals, who will provide details of any restrictions imposed on data use via data use agreements.

Field-specific reporting

Please select the one below that is the best fit for your research. If you are not sure, read the appropriate sections before making your selection.

☒ Life sciences ☐ Behavioural & social sciences ☐ Ecological, evolutionary & environmental sciences

For a reference copy of the document with all sections, see nature.com/documents/nr-reporting-summary-flat.pdf

Life sciences study design

All studies must disclose on these points even when the disclosure is negative.

Sample size	The study analyzed 644,029 confirmed COVID-19 cases in NYC and their reported 779,011 close contacts. No sample size calculation was performed as we used all available records in the contact tracing data. As the data covers over 95% of all confirmed or probable COVID-19 cases during the study period, the sample size is sufficient to capture the transmission of SARS-CoV-2.
Data exclusions	No data were excluded in the study.
Replication	Each analysis in the manuscript was performed at least three times to confirm reproducibility of the results.
Randomization	This study is a descriptive data analysis, not a randomized trial. It does not involve experiments and data collection. Not applicable.
Blinding	This study is a descriptive data analysis, not a randomized trial. It does not involve experiments and data collection. Not applicable.

Reporting for specific materials, systems and methods

We require information from authors about some types of materials, experimental systems and methods used in many studies. Here, indicate whether each material, system or method listed is relevant to your study. If you are not sure if a list item applies to your research, read the appropriate section before selecting a response.

Materials & experimental systems

n/a	Involved in the study	
☒	☐	Antibodies
☒	☐	Eukaryotic cell lines
☒	☐	Palaeontology and archaeology
☒	☐	Animals and other organisms
☐	☒	Human research participants
☒	☐	Clinical data
☒	☐	Dual use research of concern

Methods

n/a	Involved in the study	
☒	☐	ChIP-seq
☒	☐	Flow cytometry
☒	☐	MRI-based neuroimaging

Human research participants

Policy information about studies involving human research participants

Population characteristics	The study covers the confirmed COVID-19 cases and their reported close contacts in New York City living during the COVID-19 pandemic. The study population is inclusive of all genders and age groups (age distribution was reported in the paper). Race/ethnicity and socioeconomic status were not used as criteria for participants recruitment. Individual-level information on race/ethnicity and socioeconomic status was not available in the dataset. However, home locations of participants cover all zip-code areas in NYC, which indicates that participants are geographically representative and may have diverse characteristics of race/ethnicity and socioeconomic status.
Recruitment	Participants were recruited by the New York City contact tracing team according to the definition of COVID-19 infection by the US CDC. Prior to contact tracing data collection, informed consent need to be obtained from the participant, which may lead to self-selection bias. However, the overall consent rate among all index cases is over 70%, indicating that the majority of confirmed or probable cases agreed to participate. Most reported exposures were household members so there is a strong bias to household contact. We have discussed the impact of such bias on results in the paper.
Ethics oversight	Use of this dataset in this study was approved by Columbia University Institutional Review Board (IRB) AAAT2182.

Note that full information on the approval of the study protocol must also be provided in the manuscript.
